# Horse vision through two lenses: Tinbergen’s Four Questions and the Five Domains

**DOI:** 10.3389/fvets.2025.1647911

**Published:** 2025-08-14

**Authors:** Lina S. V. Roth, Paul McGreevy

**Affiliations:** ^1^IFM Biology, Linköping University, Linköping, Sweden; ^2^Faculty of Science, Sydney School of Veterinary Science, University of Sydney, Sydney, NSW, Australia

**Keywords:** animal welfare, equine behavior, equine vision, human–horse interaction, Tinbergen’s questions, Five Domains, sensory ecology, visual perception

## Abstract

To improve human–horse interactions and reduce the risk of injury, it is essential to adopt an equi-centric perspective that prioritizes how horses perceive their environment. This review focuses on the equine visual system, both because it is the most studied of the horse’s senses and because misunderstandings about how horses see can lead to unsafe or unsustainable handling. By applying two complementary frameworks, namely Tinbergen’s Four Questions and the Five Domains model, we examine equine vision from both a biological and a welfare-oriented perspective. We explore the anatomical and functional features of the horse’s eye, the development and evolution of visual capacities, and how these relate to behavior, performance and welfare, while also challenging common myths. Horses possess visual adaptations that enable them to perceive fine details, detect color, and see in dim light conditions. However, their evolutionary history as a prey species has shaped them to be highly sensitive to unfamiliar shapes and movements which we also need to be aware of. Ultimately, a deeper understanding of how horses process visual information can help correct misunderstandings, guide safer management practices, and support more ethical and effective care.

## 1 Introduction

For our interactions with horses to be safe for both parties, it is critical that we adopt a so-called equi-centric view that helps to reveal how horses perceive their surroundings. By doing so, we can understand what stimulates them and anticipate their behavioral responses. Given the current frequency of horse-related human injuries, this approach is needed and could significantly reduce the prevalence of accidents. In most developed countries, horses (usually because they are fearful) kill more humans than any other verterbrate (1). The behavior of the horse is closely linked to its perception and various sensory abilities, as outlined by Rørvang et al. (2). In this review, we aim to summarize the visual sense of horses, not only because it is the sense for which we currently have the most details, but also because equine behavioral responses, often linked to their visual perception, can pose a risk to personnel or result in horses being unjustly blamed for misbehavior. The lack of a full appreciation of how horses may respond to possible visual threats may lead to horse carers, guardians and trainers reprimanding their horses for reactions to stimuli that are not apparent to personnel. Misunderstandings about horses’ behavioral responses could lead to undesirable, and even dangerous, human-animal interactions and compromise our relationship with horses. In this review, we aim to provide a commentary on equine visual abilities, which will help to dispel common myths and address misunderstandings while offering more plausible explanations for horse behavior. We structure our approach based on the anatomy of the equine eye, its comparative position, the impact of specific anatomical features on various visual abilities under different light intensities, and the path of light as it travels through the pupil to the retina.

We examine aspects of equine vision through two lenses: one biological—Tinbergen’s Four Questions (3), and the other welfare-focused—the Five Domains (4). Our central premise is that the two lenses complement one another and applying both in parallel ensures that little of consequence is overlooked. This approach has been used recently to explore chewing in canids (5). Accordingly, we shall begin with a brief explanation of these two perspectives.

Of Tinbergen’s Four Questions, two aim to explain biological traits from a *proximate* perspective, addressing both the mechanisms underlying a behavior and its development across a lifetime. The first question (T1: Causation) investigates the immediate mechanisms, such as a visual stimulus and how that it is perceived, to trigger, for example, a locomotory response. The mechanistic attributes of visual triggers are the primary focus of the current review that goes on to include genetic predispositions among equids, some of which can arise from artificial selection. The other proximate perspective (T2: Ontogeny) considers how traits or behavioral responses develop throughout an individual’s life, for instance, how vision develops in foals or how a horse learns from experience.

Tinbergen’s two *ultimate* questions address the evolutionary reasons that underpin a trait. Specifically, they examine how a behavior or, in the case of this review, a visual capability enhances an animal’s survival and reproduction (T3: Function). The fourth question (T4: Phylogeny) seeks to explore the trait in related species to understand its place in the context of evolutionary history. Together, these four questions form the core of the biological lens through which our review will analyse vision in equids. Hence, each heading we use includes subheadings examined through Tinbergen’s lens, to address the different perspectives on the horse’s visual abilities.

Our review will then explore the implications of optimal and sub-optimal vision for domestic equine welfare within the framework of the Five Domains. This model considers four physical domains: (D1) nutrition and hydration; (D2) physical environment; (D3) health; and (D4) behavioral interactions with the environment, conspecifics, and humans. These four domains collectively influence the animal’s mental or affective state (D5) which, in turn, reflects its overall welfare status. The framework provides a structured approach to evaluating how each domain contributes to the animal’s overall welfare.

Our two lenses are united by telos, the prioritized behavioral needs left by evolution (6) We recognize that the telos of a species determines how it detects potential harm, fears it and avoids it, with an end-goal of being fit enough to reproduce.

## 2 Visual field and the implication for depth perception

**Figure 1:**
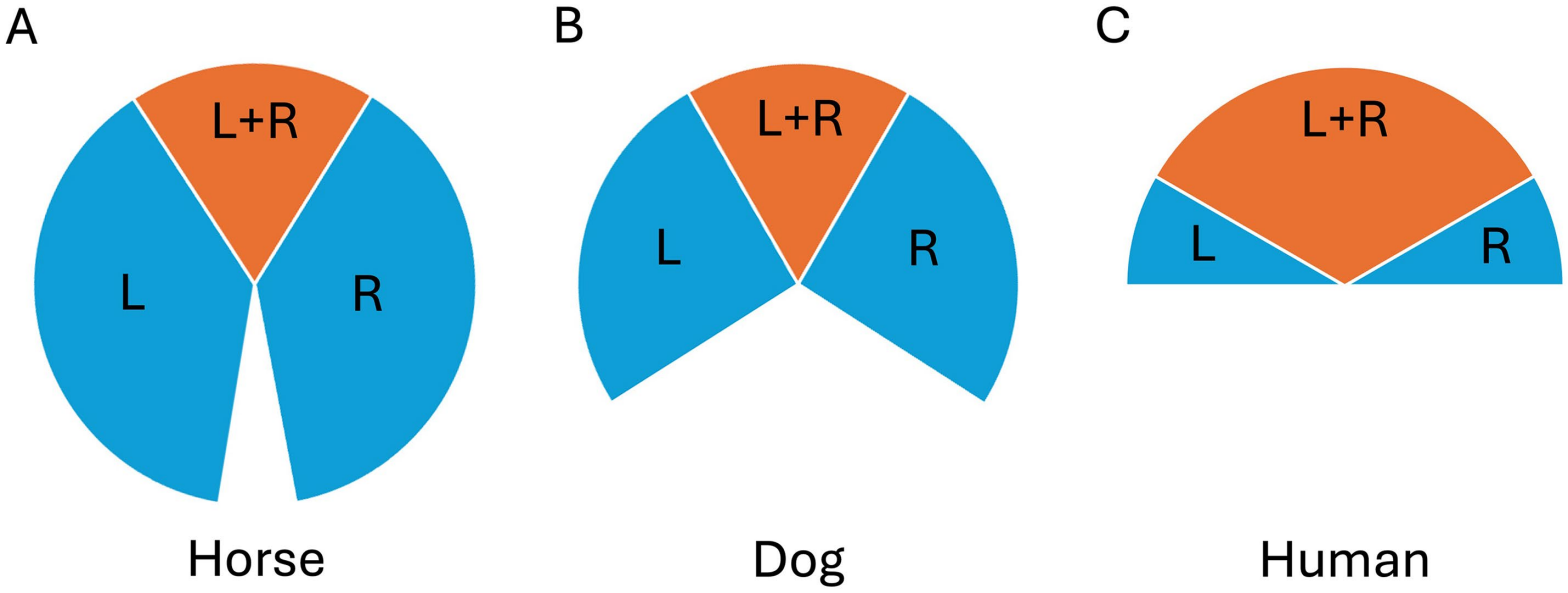
Visual fields of a mesocephalic **(A)** horse, **(B)** dog and **(C)** human, where blue indicates the left (L) and right (R) monocular fields and orange indicates the binocular field (L + R).

As an herbivorous prey animal that evolved grazing in open fields for millions of years, the horse has a visual system well-adapted for optimal panoramic awareness of its surroundings. Its eyes are positioned high on the head, clear of the much of the sward, with lateral placement that enables near-complete peripheral vision (7) (Figure 1). The only blind spots are those created by the horses’ own body structures both behind the eyes and immediately in front of the forehead. The breadth of these blind spots depends to some extent on the breed or type of horse and the concomitant placement of the eyes. They can be countered by head movements and social referencing in other members of the herd that indicate where to look when threats are detected. The combination of eye placement and extraordinary peripheral vision in a highly social species demands considerable effort on the part of predators to take a herd of horses by surprise, at least in an open field.

Despite their laterally placed eyes, horses have a 65–80° frontal overlap between their visual fields. Interestingly, this is in the same range as that of the predatory canids, drawing particularly relevant comparisons (7, 8, 155, 156) (Figure 1). When the visual field overlap and the two retinae provide the brain with slightly different information (binocular disparity) about the surroundings, depth perception becomes possible. Equine sensitivity to depth cues has been demonstrated experimentally by Timney and Keil (9), who used visual depth illusions to reveal that horses use binocular depth cues.

### 2.1 Tinbergen’s lens

In relation to Tinbergen’s questions, T3 (Function) is addressed when one considers how a panoramic view of horses’ ancestral surroundings enhanced their ability to detect predators, thereby increasing biological fitness. This is similar to other ungulates (T4 Phylogeny), emphasizing the evolutionary significance of vision in optimizing herd surveillance. In addition, the ability to perceive depth facilitates rapid navigation of unfamiliar and uneven terrain and assessment of obstacles, both of which are crucial for survival for flight-prone prey animals, such as horses (10). More saliently, fleeing as a group depends on synchrony and keeping group members in sight. Conspecifics to the sides and to the rear provide critical information about predatory threats that only they can see, while those in front help steer the group through the terrain ahead.

As horses have evolved, the terrain that equids and their ancestors have preferred has undergone change. Notably, early dog-sized horses, such as the so-called dawn horse *Hyracotherium*, inhabited forested environments where they foraged on soft vegetation, including browse in the form of buds and leaves. This is an example of how the challenges of the environment may underscore the importance of depth perception in the early evolutionary history of horses (T4 Phylogeny), even though they later adopted a feeding strategy for open plains that involved more roaming behavior and grazing (11). The ancestral vegetation provided places to hide but browsing in thorny vegetation runs the risk of penetrating ocular injuries, an outcome that is only partially offset by having vibrissae around the eyelids to detect the presence of solid objects.

T1 (Causation) considers the mechanisms that underpin, for example, predator discrimination which may not be limited to the extensive visual field and depth perception but could also involve both spatial and temporal resolution. Here we note horses’ visual ability, in combination with input from other sensory systems, to detect fine details and the movement of salient respectively, topics to which we will return.

In sheep, it has been suggested that the mother-infant bond is crucial for the development of depth perception, since mother-reared lambs consistently avoided the perceived drop sooner than isolated lambs [T2 Ontogeny; (12)]. When considering depth, perception may be further refined through experience (T2 Ontogeny). For example, when familiar trees (of known size) appear smaller than when directly in front of the horse, individuals learn that they are at a greater distance, which then serves as a mechanism for providing depth perception (T1 Causation). Another example of a depth cue is optical expansion, which arises when an approaching object appears to grow larger in a predictable, non-linear manner, as dictated by physics. Our own brains subconsciously interpret these cues, when we apply an innate understanding of physical principles and have appropriate experience. Currently, little is known about how non-human mammals process such depth cues. Nevertheless, we know that depth information is not restricted to only frontally eyed animals (9, 156); and, until evidence suggests otherwise, we should assume that equine brains are also capable of accurately perceiving depth and relative distances in their surroundings.

### 2.2 It is enough for the horse to see with only one eye

Our commentary is, not least, designed to examine some abiding myths, common among horse people, about equine vision. An example of such myths (Table 1) is the belief that if a horse sees an object with only one eye, “the other eye has not seen it.” This assumption is commonly used to explain why horses react with apparent naivety, to a given object, such as an unfamiliar garbage bin, when passing it for the second time but from the opposite direction to the first pass. Similar observation was made by Corgan et al. (13) who reported on a study in which 20 horses passed a children playset multiple times during habituation, yet still exhibited strong reactions when approaching the same playset that now had been rotated 90 degrees. There is no lack of decussation [the crossing of nerve fibers in their corpus callosum; (14)] that would explain horses reacting with apparent naivety to the garbage bin again in the reverse direction. Indeed, behavioral studies by Hanggi (15) clearly demonstrate that horses transfer visual information between the brain hemispheres. In her experiment, horses (*n* = 2) were trained to perform several visual discrimination tasks while one eye was blindfolded. When the blindfold was switched to the other eye, the horses continued to discriminate at a high level, providing strong evidence of interhemispheric communication and effectively debunking the myth that each eye processes visual information in isolation. It is important to note that, for this type of conceptual study, a small number of horses is sufficient to demonstrate the underlying principle.

**Table 1:** Summary of common myths and misunderstandings.

Myth	True/false	Explanation	Research on horses
The horse, with its laterally positioned eyes, has poor depth perception	False	Depth perception can be achieved with either overlapping visual fields (which horses have, similar to dogs and yet no one typically claims that dogs have “poor” depth perception) or through monocular cues using one eye	Timney and Keil (9) revealed that horses (*n* = 2) can perceive depth illusion in photographs, and they also found that horses trained in stereopsis tests with both eyes performed better than those trained with one
If a horse sees an object with only one eye, “the other eye has not seen it”	False	The visual cortex receives and processes information regardless of which eye detects an object	Hanggi (15) demonstrated that horses (*n* = 2) trained on a discrimination task with one eye blindfolded, performed equally well when the blindfold was switched to the other eye
The horse requires much longer time than humans to dark adapt	False	Rod adaptation time is similar across various mammals	Ben-Shlomo et al. (38) showed through ERG studies (*n* = 6) that horses are fully dark adapted after 20 min. The horses (*n* = 7) in Ignacio et al. (39) were dark adapted after 16 min
The horse has “poor visual acuity”	False	The ganglion cells in the horse’s eye, which are indicative of visual acuity, are densely packed along a horizontal streak across the retina	Timney and Keil (45) found that the best-performing horse in their study (*n* = 3) could see a detail at 25 m that a human could see at 30 m. Also, breed differences may arise from retinal differences among morphotypes (42)
The horse cannot see colors	False	Horses are dichromatic and can perceive blue to green-yellow colors. Red is probably perceived as a dark green color	Grzimek (74) was the first to fully demonstrate color vision in horses and Roth et al. (23) showed that they perceive a continuous scale of colors, with gray or bluegreen color at the neutral point (*n* = 3)
The horse is very good at navigating in extremely dim light conditions	True	The horse has several adaptations in the eye that support vision in dim light conditions	Hangii and Ingersoll (157) showed that horses can easily navigate in a test arena and distinguish different shapes at dim light conditions, even when the human operators could not see anything (*n* = 4)

### 2.3 Tinbergen’s lens

One possible explanation for reports of this apparent naivety is that, on the return journey, the garbage bin appears within a different visual context because the background imagery changes when passed in different directions (T1 Causation). For a prey animal, it is important to be cautious even about minor changes in the surroundings as they might provide visual hints about a potential threat (T3, Function). As an example from another genus, Tammar wallabies (*Macropus eugenii*) that lived without predators for thousands of years still respond with antipredator behaviors, such as vigilance and alarm calls, when presented with a crude visual predatory model (16). Hence, many antipredator responses can be genetically hardwired (T1 Causation and T4 Phylogeny) even if no predators are present (16, 17). Returning to the “garbage bin” reaction; another possible explanation of the apparent asymmetry in the horse’s response could also be linked to lateralisation of emotions, because research shows that horses react more strongly when observing a novel object with the left eye [(18, 19): but see also Baragli et al. (20)]. We will explore this further under the 4th Domain subheading. Additionally, one should not forget that the horse’s other senses (T1 Causation), such as olfaction, may also play a role in surveillance, especially considering factors such as wind direction, temperature and odor plumes, which could make the object seem unfamiliar or more salient when approached from a novel direction.

## 3 The pupil adaptation to different light intensities

**Figure 2:**
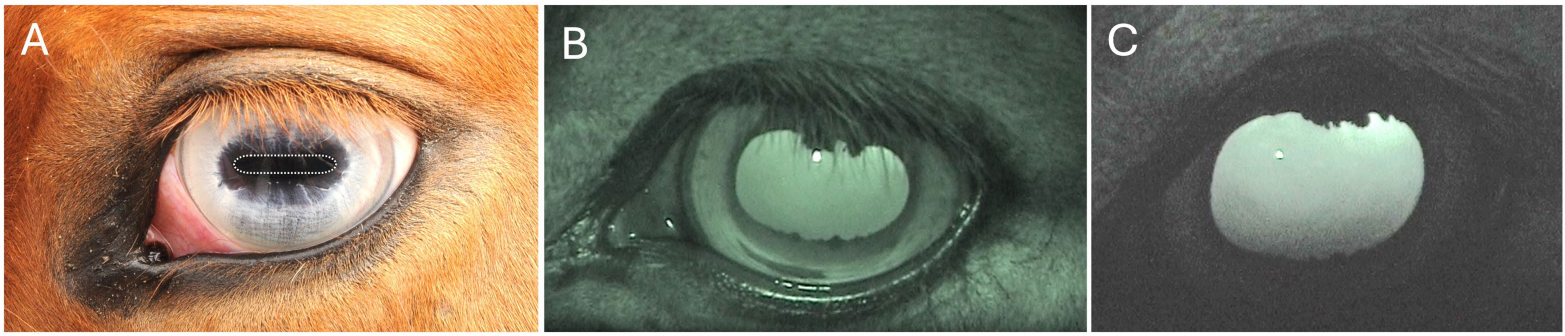
The horse’s eye **(A)** on the left shows a fully constricted, light-adapted pupil (dotted line indicates the pupil boundary, shaped as a horizontal ellipse). The **(B)** middle and **(C)** right images are from different horses, showing dark-adapted, dilated pupils with the corpora nigra visible in the upper and lower margins of the pupils. Photos: Lina Roth.

Horses are normally arrhythmic and active during both day and night (21, 22). So, they face the challenge of requiring vision both in bright and dim light intensities and thus rely on pupils that can quickly modulate the amount of light reaching the retina. As dawn breaks and light becomes abundant, the equine pupil constricts into a horizontally elongated aperture (see Figure 2). In addition to the constriction of the pupil, extensions of the iris form the *corpora nigra* which, in bright light, enlarges over the pupil, like an awning and shields the retina from the strongest daylight.

In low light conditions, the retina requires relatively more light to generate a signal strong enough to override the natural random electrical activity in the photoreceptors (known as background noise), allowing for the detection of contrasts (158, 159). Notably, moonlight can be a million times dimmer than sunlight, so the more photons the eye can capture under such nocturnal conditions, the better. Accordingly, as light levels drop, the pupil of the horse dilates, allowing more light to enter the eye (Figure 2). The pupil dilates into a large oval-circular shape, reaching up to approximately 30 mm in maximum diameter (23), which has roughly seven times the area of the constricted equine pupil in sunlight and is about six times larger than that of a fully dilated human pupil with a maximum diameter of 8 mm.

### 3.1 Tinbergen’s lens

In relation to Tinbergen’s questions, specifically T3 (Function) and T4 (Phylogeny), the horizontal shape of the horse’s pupil is shared among many other herbivorous prey mammals that occupy similar ecological niches, as it enhances panoramic vision and helps predator detection and survival. In contrast, predators have circular or vertical slit pupils (24). Horizontal slit pupils allow for greater control over the aperture area compared to round pupils, and are especially beneficial for animals active across a broad range of light conditions, such as the horse. Also, while vertical slit pupils enhance distance judgment in ambush predators, the horizontal slit pupil, with a reduced vertical extent, increases the depth of field for the horizontal view which helps maintaining a sharp image across varying distances (24). Hence, the horizontal slit pupil improves image quality and supports a panoramic horizontal view, aiding in the detection of approaching predators.

The mechanism behind the pupillary response (T1 Causation) is primarily controlled by the autonomic nervous system, specifically the pupillomotor reflex which, during constriction, can be triggered both by light on the retina and, in many mammals, by direct illumination of the iris muscles surrounding the pupil (25). The pupillary response in mammals is rapid, typically occurring within 1 s, for example circa 133–381 msec in dogs, 299 msec in rats, and 180–500 ms in humans [see review (25)]. Of note, this response is influenced not only by light intensity but also by the spectral quality of the light stimulating specific regions of the eye, and preceding dark adaptation. Although data on the dynamics of pupillary responses in horses (or other ungulates) are limited, they would be unlikely to differ much from other mammals. That said, what has been observed in horses is that pupillary responses are affected by massage therapy (T1 Causation), with the pupil size decreasing with the activation of the parasympathetic nervous system that accompanies rewarding tactile contact [(26); *n* = 12]. The role of mutual grooming in horses as a form of social bonding (27) aligns with this evidence from humans applying massage. Indeed, humans grooming the withers can reduce horse heart rates (28) and modulate oxytocin (29) as conspecifics do (30). In contrast, twitches applied to the nose of horses, for restraint purposes, that may operate through the mediation of endorphins released in response to pain (31), induce a pupillary dilation [(32) *n* = 14]. This is similar to humans where pupil dilation occurs, irrespective of light condition, when exposed to pain (33).

## 4 Retinal adaptations to different light intensities

To detect the magnitude and range of light (as described earlier), the horse retina, just as in most vertebrates, contains two types of photoreceptors: rods and cones, which are specialized for different light intensities (34, 163). Of these, rods are generally the more light-sensitive. They can respond to single photons and are primarily used for vision in dim light conditions when photons are sparse (164). Cones function at higher light intensities and may enable color vision (to which we will return later). Depending on the part of the diurnal cycle when the species is most active, the composition and ratio of rods and cones varies. That said, rods typically outnumber cones in most mammalian retinae. In humans, for example, cones comprise less than 5% of photoreceptors (35).

As light intensity increases, the cones are activated. As light intensity increases further, the photopigment in the outer segments of the rods gradually become bleached, transiently rendering them unable to respond effectively. To regain sensitivity in low-light conditions, the rods require time to adapt. In nature, this is normally not an issue because light intensity changes gradually.

### 4.1 Tingergen’s lens

The evolutionary adaptive value (T3 Function) of having photoreceptors sensitive to different light conditions is again probably linked to predator detection and survival to which we will return when describing the mammalian evolution of photopigments (under the color vision section below). Having a retina with both rods and cones is typical for most vertebrates (T4 Phylogeny) and is missing only in those species with long ancestral history of strictly diurnal activity [such as lizards, that only have cones; (36)]. Considering T2 (Ontogeny), the differentiation of rods and cones occurs during embryonic development so, at birth, foals already have a highly functional retina with both rods and cones.

Interestingly, a widely circulated myth suggests that horses take much longer than humans to adapt to darkness, also known as to dark adapt [e.g., recently in (37)]. However, studies using electroretinography (ERG), which measures the eye’s electrical response to brief, dim flashes of light, reveal that the photoreceptors of horses (*n* = 6) start adapting within 5 min and are fully dark-adapted and responsive after just 20 min, with no significant changes beyond that period (38). The same is seen in many other mammals (T4 Phylogeny), including dogs, rats, and humans (160–162). Notably, a recent ERG study revealed that dark adaptation in horses is complete after only 16 min [(39); *n* = 7]. The misconception about an unusually long dark adaptation in horses may arise from their behavioral response to sudden and large changes in light intensity, (such as stable lights being turned off), rather than from actual dark adaptation at the level of their retinae.

## 5 Retinal topography and visual acuity

Once the visual signal has activated the photoreceptors, it is further processed by bipolar and horizontal cells in the retina. Contrasts are enhanced and the signal is made less dependent on overall light intensity (159). Before the signal exits the eye, it reaches the ganglion cells, whose axons then transmit it to the brain for further processing and conscious perception. The distribution of ganglion cells differs throughout the retina, being denser in the area where the need for most visual information peaks. Hence, the density of photoreceptors and ganglion cells provides an indication of the eye’s spatial resolution and species-specific purpose.

The highest resolution is achieved when each ganglion cell receives signals from only a few photoreceptors (and sometimes even only one). Typically, this type of connection is confined to a few small areas of the retina. In human retinae, this region is called the fovea, in the middle of which the ratio of photoreceptor signal and ganglion cells can reach 1:1. This ratio provides the most detailed information and thereby highest spatial resolution (163). However, in most regions of the (human) retina, ganglion cell density, and thus spatial resolution, is much lower, but, as you might have noticed, our brain is amazing at filling in missing information. Indeed, we do not typically think about how low our spatial resolution is in our peripheral vision. Nor do we notice that we have a blind spot where the optical nerve (containing all ganglion axons) exits the eye. Instead, brain processing ensures that we remain unaware of this limitation by bridging the gaps to create a seamless and continuous perception of our environment [e.g., (40, 41)].

**Figure 3:**
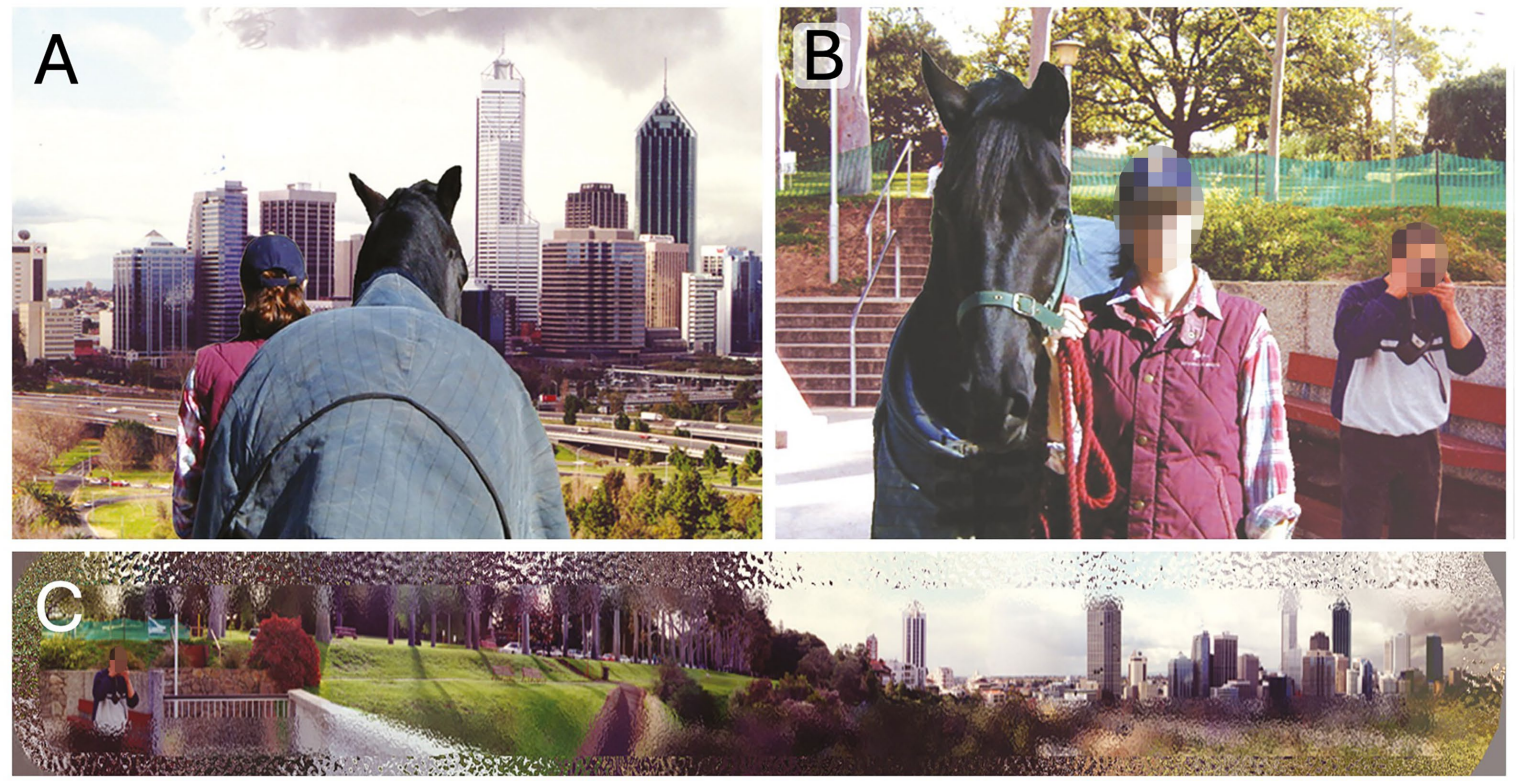
The **(A)** rear view and the **(B)** front view when standing looking out over Kings Park (Perth, Western Australia), where the **(C)** horse, with its panoramic view with highest resolution along the horison, can both enjoy the view and see the man behind them taking a picture. Source: Photos copyright Alison Harman, reproduced with kind permission.

In humans, the most central part of the fovea contains only cones (35). So, in dim light conditions, when rods are active, the fovea functions as a small blind spot. However, as we have just acknowledged, this limitation usually goes unnoticed except when trying to focus on a tiny faint star in the night sky. For horses, the ganglion cells reach their highest density in a narrow, horizontally oriented streak, thus allowing for best resolution along the horizon (7, 42–44) (Figure 3). In addition, a slightly higher, but fairly uniform, density is found in the temporal region of the horizontal streak. In contrast to the central part of the human fovea, the horizontal streak in horses contains both rods and cones and thus permits highest resolution in both bright and dim light intensities, even though the resolution in dim light would be lower (as explained below). Hence, the horse eye is well adapted for an arrhythmic lifestyle under varying light conditions.

In daylight conditions, Timney and Keil (45) found that horses (*n* = 3) had an average visual acuity of approximately 20/30 on the Snellen scale. Comparatively, this means that a “normal human” can see at 30 meters what a horse can see in the same detail at 20 meters. However, the best equine acuity obtained in that study was 23.3 cycles per degree which would equate to 20/25; and as such is very close to our own visual acuity. Therefore, if a human with normal vision (20/20) distinguishes a certain detail at a distance of 30 meters, the best of the three horses Timney and Keil (45) studied would see the same detail at 25 meters distance. Notably, the inter-species differences are not as considerable as was once generally thought. This is also reflected in behavioral experiments in which horses could discriminate between open and closed eyes in two humans from several meters away (46).

### 5.1 Tinbergen’s lens

Considering Tinbergen’s question on the principal adaptive value (T3 Function) of high visual acuity across the horizon, this trait is again primarily associated with predator detection in ungulates, but may also play a critical role reading the signals the rest of the herd is constantly emitting as they encounter appetitive and aversive stimuli. The horizontal visual streak is widespread among terrestrial mammals [T4 Phylogeny; (47)] and both terrain and body size of the viewing animal have been linked to variations in retinal topography contributing to successful detection (47–49). In mammals with laterally placed eyes, a temporal shift in the area of highest resolution often directs peak aquity toward the forward field of view (47) which is also true for the horse, as previously mentioned (7). Additionally, taller species, such as the giraffe (*Giraffa camelopardalis*) and the camel (*Camelus ferus*), show extended temporodorsal regions of high cone density (49). Interestingly, this distribution is not exclusive to tall species as small montainous species, such as the Barbery goat (*Ammotragus lervia*), show a similar dorsal temporal extension (49), and sheep [*Ovis aries*; (50)] may also possess a relatively high-density area in the dorsotemporal retina, suggesting that retinal topography reflects complex interactions between habitat, body size, and phylogeny. Additionally, when Harman et al. (51) studied the ganglion distribution in dromedary camels (*Camelus dromedarius*) they found two regions of high density located in temporal and nasal regions of the retina, both with pronounced elongations along the vertical axis. A similar pattern has been found in African elephants (*Loxodonta Africana*), with the temporal region specialized for acquiring the highest visual aquity, likely from the areas associated with trunk movement, such as during foraging (52). Hence, while taller ungulates and mountainous species may have a distinctly vertical distribution of cells across their retinae, and, in some cases, even dual high-acuity regions, the horse possesses a horizontal streak that is relatively narrow along the vertical axis, incorporating a temporal area with the highest visual acuity (7, 42–44).

For horses, the importance of visual communication among the herd may be reflected in their relatively high visual acuity. By discriminating body language, facial expressions and ear positions of conspecifics (53, 54), horses can minimize their individual risk of unnecessary injuries due to agonistic interaction. Indeed, social communication and interaction are vital and have been shown to have direct fitness benefits in free-ranging horses [T3 Function; (55)]. Interestingly, behavioral assessments suggest that visual acuity in other ungulates (T4 Phylogeny) is much lower, with camels being able to discriminate 10 cycles per degree (51) and cattle between 2.6–5 cycles per degree (56, 57). This may reflect the relative mobility and resultant expressiveness of the equine face. It may also reflect ways in which interactions with conspecifics (and others) vary with their species-specific behavioral time budgets. While horses graze for most of the day, continuously communicating and keeping track of conspecifics as well as potential threats, cattle and camels spend less time foraging, dedicating much time ruminating and resting. Indeed, in cattle, a greater part of the daily total rumination (reported to range between 5 and 10 h) is performed lying down and they also tend to lie down more while resting as opposed to standing (58).

Hence, the notion that horses have “poor visual acuity” can be considered a myth (Table 1), especially when equids are compared to other ungulates. The spatial resolution of each species is adapted to their evolutionary needs, just as our resolution is adapted to our needs, and just as eagles’, whose acuity can reach 150 cycles per degree, are adapted to theirs (159). If an eagle were aware of human visual capabilities, it might be the one to scoff. Of course, we do not perceive our own vision as “poor.”

### 5.2 Domestication and artificial selection might influence visual abilities

When we consider differences in vision between species, we should not overlook the prospect of smaller differences between morphotypes within a species. Of note, there are indications that there could be breed differences among horses, which indicate that selective breeding may have, however inadvertently, affected visual abilities in horses. Evans and McGreevy (42) found a strong correlation between ganglion cell density in the horizontal streak and skull length when studying the Arabian horse (shortest skull), Thoroughbreds (mid-length skull), and Standardbreds (longest skull). However, potential breed differences in visual acuity have not yet been tested behaviorally. In dogs, where associations between skull shape and ganglion cell distribution have also been found (59), there are indications from behavioral experiments studies of individuals, although few (*n* = 6), that short-nosed dogs may have higher visual acuity (24 cycles per degree) than other dogs (60).

Hence, there could be breed differences in visual acuity among morphologically diverse domesticated animals and future studies could explore the practical implications of this. Until they are completed, we should not assume that all morphotypes within a species perceive the world the same way. To do so risks poor performances, arising from different perception, being blamed on the animals’ lack of motivation or even lack of cooperation; both of which can lead trainers becoming frustrated and considering the escalation of force and coercion.

## 6 Color vision

As Newton discovered in the 17th century, light itself is not colored (61). Color is created only when light is subjectively perceived by an observer with color vision. Hence, color exists only in the brain and the observer’s experience.

**Figure 4:**
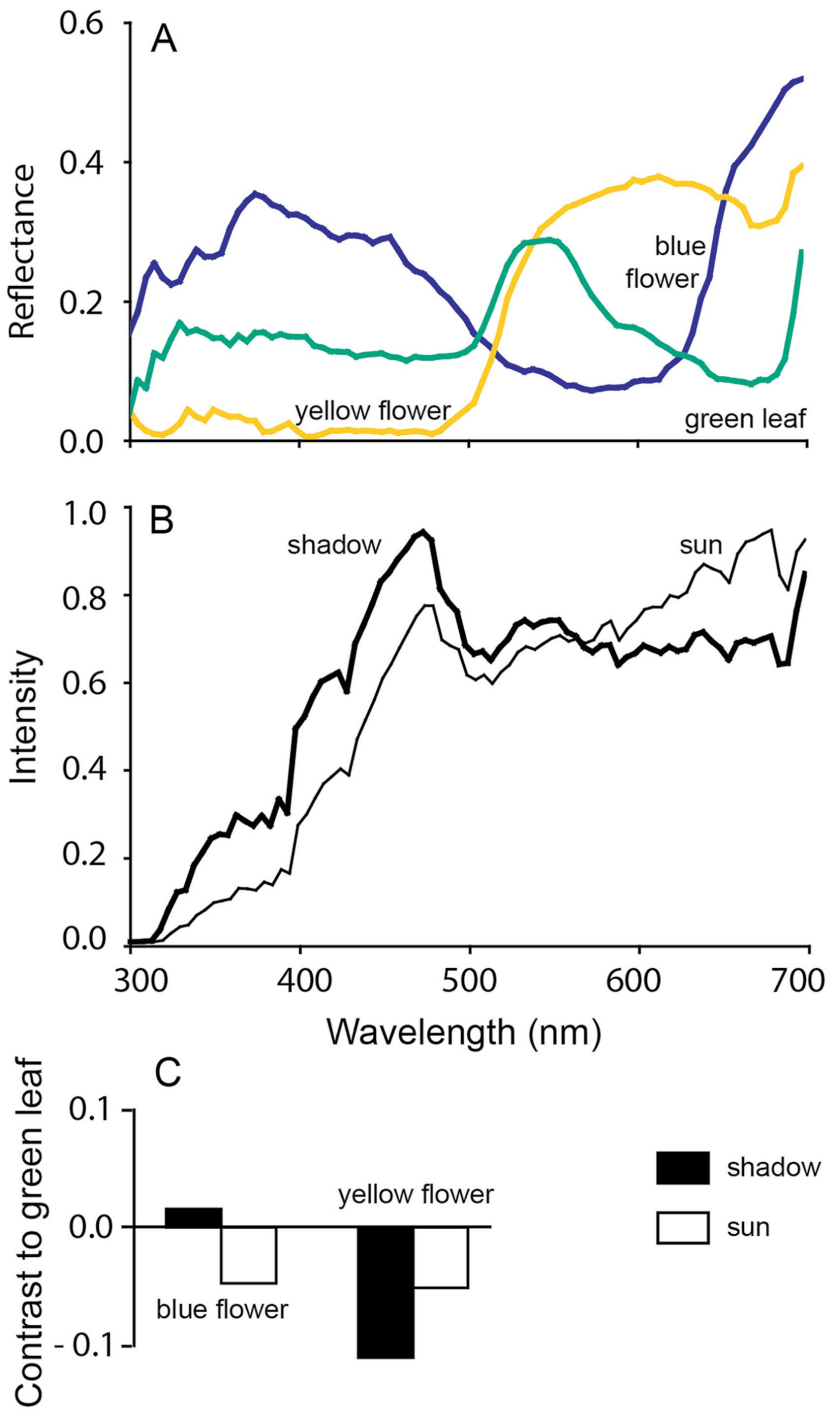
Achromatic contrasts under different light conditions. The **(A)** reflected light from a typical yellow flower, a blue flower and green leaves differs in spectral composition (300–700 nm). However, **(B)** the spectral composition of direct sunlight and skylight in shadow, also differs. This results in **(C)** changes in achromatic contrast between the flowers and leaves when viewed by [only] a “green-sensitive” fotoreceptor. In sunlight, both flowers show similar contrast against the green leaves, whereas under shadow, the contrast differ. When color vision is used instead, the contrast remains the same regardless of illumination conditions. Reproduced with kind permission from Kelber and Roth (64).

Each single photoreceptor in the retina will count [only] the number of absorbed photons and thus will not be able to generate a color signal on its own. For an animal to have color vision, it must possess at least two types of photoreceptors operating across similar light conditions. These photoreceptors must differ in their spectral sensitivities, and their signals must be compared further down in the retina, thereby generating a color signal (62, 63). These photoreceptors, usually the cones, must be oriented in roughly the same direction as one another, and be distributed in the retina such that their signals can be compared across subsequent cell layers to generate color information. According to a commonly used definition, color vision is the ability to distinguish between objects of the same shape, size, texture, and brightness that differ only in the spectral composition of the reflected light (62, 63). Importantly, and this may be underestimated, color vision plays a critical role in reliably discerning contrasts, particularly in environments with patchy lighting. If one considers a threshold or an edge where changes in brightness could indicate a boundary between two objects, the contrast may also be caused by shadows, and thus potentially mislead the observer. In this instance, color vision helps by distinguishing actual object boundaries from shadow-induced contrasts, enhancing the detection of food, landmarks, conspecifics or predators and improving navigation in complex environments where brightness alone may be deceptive. Similarly, color vision is valuable under different light conditions or during transitions between day and night, such as at dawn and dusk, when the spectral composition of light shifts (see Figure 4). Here, cone adaptation to ambient light helps maintain color perception and contrasts despite changes in the lighting conditions. In short, the spectral information in the light reflected from objects offers a more reliable cue than just brightness alone (36, 64–66).

### 6.1 Tinbergen’s lens

It is important to appreciate the evolution history of vertebrate color vision, which is related to T3 (Function) and T4 (Phylogeny). The basal condition for all vertebrates is to be tetrachromatic with four different cone pigments, allowing them to perceive a broad range of colors that could be used both for communication and detection of prey or predators. During the period when early mammals were thought to have been primarily nocturnal (due to the threat of predation from, for example, co-existing diurnal Archosaurs), they relied more on senses other than vision, and lost two of the ancestral four vertebrate cone pigments (36, 67, 68). Interestingly, more recent studies on the photopigments suggest that the early mammals were primarily active toward dawn and dusk, rather than at night (69), which may help to explain why mammals retained dichromatic vision, instead of losing more cone pigments. Regardless, as a result of the predation pressure (T3 Function), mammals lost the ancestral tetrachromatic color vision which still persists in fish, many birds and some reptiles, often those distinguished by vibrant colorations (63). Consequently, most extant terrestrial non-primate mammals have two cone pigments, which allow for dichromatic vision [while the third cone pigment in trichromatic primates and humans is a result of a gene duplication 30 MYA; (70)].

Plainly, the myth (Table 1) that horses, or for that matter, dogs and cats, cannot see color is incorrect. Crucially, the term “color-blind,” used to describe dichromatic human males who are typically red-green deficient, is misleading. While dichromats do perceive colors, their experience differs from that of a trichromat. The three cone pigments in trichromatic humans give rise to a two-dimensional chromatic space, enabling the perception of two qualities of color: hue, which refers to the attribute of the tint of a color, such as blue or green, and saturation, which relates to the purity of the color (71). Thus, saturation is linked to the spectral purity of a color—that is, the degree to which a chromatic stimulus differs from an achromatic stimulus, such as white or gray, regardless of brightness. An unsaturated color contains a significant amount of white or gray, whereas, for example, blue with a very small degree of gray or white is considered highly saturated. As will become evident, the dicromats experience only a unidimensional color space.

### 6.2 How is the dichromatic color space perceived

**Figure 5:**
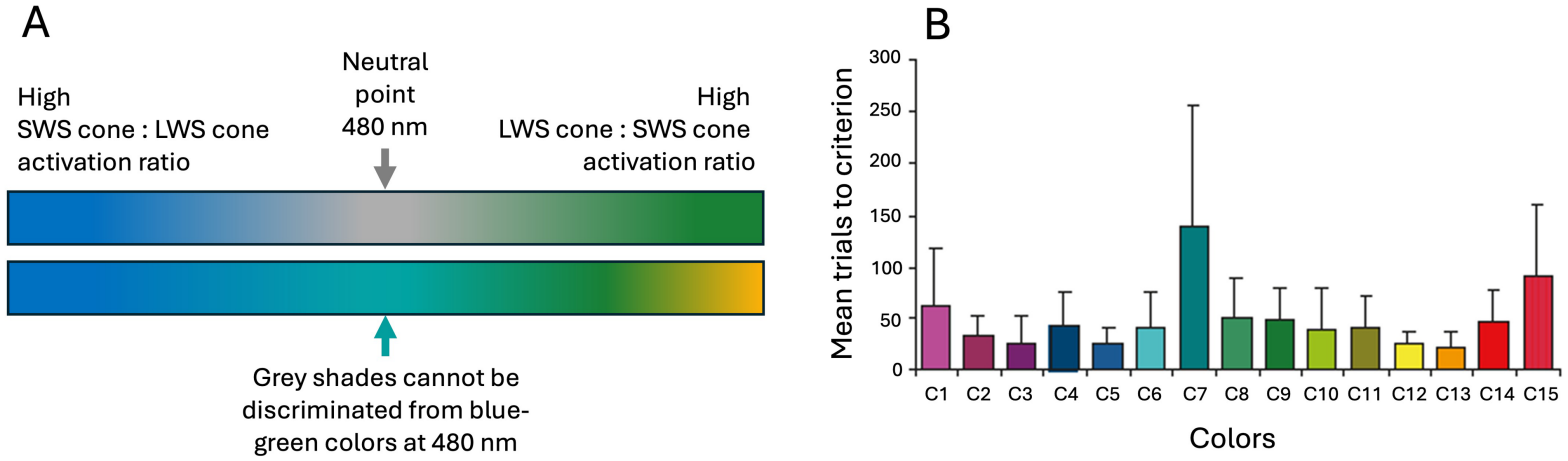
The horse’s color space ranges continuously from the **(A)** blue spectrum to green/yellow. The neutral point in the middle (480 nm) is perceived as any other color by dichromatic animals, and since colors at the neutral point generate similar cone excitation as grays this **(B)** makes it hard for horses to discriminate as shown from the very high trial numbers (mean with 95 CI) for C7 in Hall et al. (79). They trained horses to discriminate 15 colors (C1–C15) from gray which the horses did eventually for all colors, even the blue-green C7 that was probably very close to the neutral point. Note, that it remains unclear whether horses perceive the neutral point as a shade of gray or a blue-green color corresponding to 480 nm. Panel **(A)** is color space approximations by Lina Roth. Source: Panel **(B)** reproduced from Hall et al. (79), copyright American Psychological Association, with kind permission.

In simpler terms, we could refer to the dichromat cone types as the “blue-sensitive cone type” and the “green/yellow-sensitive cone type,” but the more accurate terminology would be short-wavelength-sensitive (SWS) cone type and long-wavelength-sensitive (LWS) cone type which reflects their evolutionary history and their spectral sensitivities (63, 67, 72). In the one-dimensional dichromatic color space, between colors that elicit a full response from the SWS cones (perceived as blue) and those that elicit a full response from the LWS cones (perceived as green/yellow), there is a so-called neutral point (Figure 5A). The neutral point, located at 480 nm in horses, corresponds to the wavelength of monochromatic light that produces responses in both cone types, similar to the way white and gray activate them (73). Thus, at the neutral point for a dichromat, the perception of gray shades is indistinguishable from the monochromatic blue-green light. This phenomenon may explain some of the inconsistent results from early color vision studies on dogs and horses. Because certain green and blue colors are located very close to the neutral point in the dichromatic color space, they are difficult to distinguish from gray. Indeed, behavioral studies on mammals in the early 20th century were ambiguous (68). In horses, Grzimek (74) conducted the first convincing behavioral experiments that confirmed color vision. Subsequent studies support Grzimek’s results, although there are small inconsistencies in the details about the specific colors that horses can discriminate. These probably arise from confusion about the neutral point (75–78). This prospect is further supported by the findings of Hall et al. (79) who trained horses (*n* = 6) to discriminate 15 different colors (Figure 5B). These horses successfully learned to distinguish all colors but required significantly more trials to eventually differentiate gray from a blue-green hue. Hence, the approach used by Hall et al. (79) significantly redressed misconceptions about the dichromatic color vision in horses.

### 6.3 Relative color learning in dichromats

An abiding question about the neutral point in the dichromats is whether it divides the one-dimensional color space into two color categories, or alternatively, whether dichromats perceive the color at the neutral point just as they perceive any other color, with the entire color range forming a continuum. As trichromatics, humans are accustomed to categorizing colors (and everything else we encounter) but that may not be the primary purpose of color vision for all animals, given the reliable contrast information it provides. Indeed, studies of dichromats, such as the Tammar wallaby (*Macropus eugenii*) and horses, suggest that dichromats do not necessarily categorize colors but rather perceive a color continuum (77, 80). In a two-choice study of horses (*n* = 7), this possibility was tested by training subjects to choose either the color reflecting longest wavelengths or the color reflecting shortest wavelength. This sort of study tests the so-called relative learning concept, which has previously been shown in horses using stimuli based on relative sizes (81). It emerged that horses (and Tammar wallabies) could learn colors in a relative manner (i.e., as colors relate to, and can be compared with, one another) and that they generalize both across their neutral point as well as between colors on the same side of the neutral point (77). The horses could even discriminate between different yellow and green colors, a challenge that has previously yielded ambiguous results. Hence, contrary to the suggestion by Jacobs and Deegan (82), it may be that the neutral point does *not* divide the dichromatic color space into two categories. Instead, it seems that horses may treat the colors/grays at the neutral point in the same way as they do any other color they can perceive. Regardless, we still do *not* know what the horses perceive at the neutral point. In behavioral tests, we can observe only that they do not discriminate between gray shades and the color corresponding to their neutral point located at 480 nm (73).

So, a dichromatic color space has one less dimension than the trichromatic one. While humans perceive both hue and saturation, a dichromat may perceive blue and green as a continuum of saturation, with the lowest saturation (appearing most white or gray to us) being around the neutral point. Alternatively, they might perceive a continuous shift in hues, from blue to blue-green at the neutral point, and then to green and yellow (Figure 5A). Either way, chromatic cues remain more reliable than achromatic information in the environment across various light conditions as previously discussed (Figure 4).

## 7 Dim light vision—optical and neural solutions

Seeing well in dim light places considerable demands on the visual system. As light intensity decreases, photons become increasingly scarce. As mentioned before, an optical adaptation to dim light intensities is to be able to dilate the pupil (and have a large eye), allowing more light to enter the eye (158, 159). An additional optical structure that increases the absorption of rare photons in dim light in some species, including the equidae, is the tapetum lucidum. The tapetum consists of reflective cell layers that function as a mirror at the back of the eye. It is this structure that causes the eyes of some animals to glow when illuminated by powerful lights at night. The tapetum gives the photoreceptors a second chance to capture non-absorbed photons, thereby enhancing the sensitivity of the eye (159), though this advantage comes at the cost of some light scattering, which may reduce visual acuity. Nevertheless, a tapetum greatly improves vision in dim light and is found in various vertebrates active in dim light conditions, although the composition and location of reflective cells within it vary which can result in differences in the eye-shine (83, 84).

In addition to optical mechanisms and receptor adaptations, neural adaptations can also enhance sensitivity. One neural mechanism to improve the signal-to-noise ratio in dim light is to sum the signals from several neighboring photoreceptors. As a result, the ganglion cells receive a much more reliable signal than from single photoreceptors, although the trade-off is a loss of spatial resolution, which can be thought of as fewer but larger pixels in the image (85, 159). Another neural strategy to improve vision in dim light is to sum the signals over time; which, to use an analogy from camera specifications, is similar to a longer exposure time in a camera. This results in a brighter image, but motion becomes blurred as the integration time increases (86). Animals that need to see well in dim light often benefit from sacrificing both spatial and temporal resolution, with the extent of this trade-off varying according to their lifestyle and visual needs.

In their investigation of dim light vision, (157) trained four horses to discriminate between different two-dimensional shapes, such as triangles and circles, at various light intensities. Their horses navigated with ease in very dim light conditions corresponding to very dark moonless night (<6.81E-05 cd/m^2^) such that even the dark-adapted test personnel could not see the horses as they undertook the task. To give a sense of how dark conditions were, in order for the cameras (with night vision) to record the horses’ choices, the researchers had to adhere reflective strips on the head of each horse. This study showed that horses can readily distinguish different shapes at light conditions similar to dim moonless nights. Hence, it supports the notion that horses are very good at navigating in extremely dim light conditions.

It has been hypothesized that, with their arrhythmical lifestyle, their very large eyes, and tapeta lucidum, horses might be able to use color information in dimmer light than humans can (23). As mentioned before, during dusk and dawn, changes in light conditions make color information more reliable than achromatic contrast cues for distinguishing objects, predators, or other relevant stimuli. That said, in dim light, a limitation of color vision emerges when cone signals are compared with one another to generate a color signal. As daylight fades, photoreceptor noise accumulates, while the actual signal remains weak, reducing the effectiveness of color vision in dim light. Nevertheless, since dichromatic color vision involves comparisons of only two cone types, it should be more efficient and less noisy than trichromatic color vision in dim light (before the rods fully take over). As a result, dichromacy would, at least theoretically, allow for better stimulus discrimination in low light conditions when the signal is weaker and noise relatively lower than in trichromatic or even tetrachromatic color vision (87). Thus, dichromacy might also have been beneficial during the nocturnal (or dawn and dusk) phase of early mammalian evolution.

Notwithstanding the above, when Roth et al. (23) trained three horses to discriminate between different shades of blue and green at varying light intensities, ranging from day light intensities to starlight, the horses’ performance was comparable to that of humans. Although there were some individual differences, all horses chose the correct stimuli as illumination decreased to moonlight intensities (23). Importantly, both human and equine color vision continues to function across the significant changes in illumination that occur during dusk and dawn. As illumination decreases still further, the more light-sensitive rods take over. Hence, color vision is not only about how many hues can be discerned but also about obtaining more information from the surroundings, even when the light condition changes.

### 7.1 Tinbergen’s lens

To repeat, prey animals benefit from good vision in dim light to detect threats from potential predators and warnings from conspecifics (T3 Function). Indeed, large eyes capable of capturing scarce light at night are common among ungulates (T4 Phylogeny), as is the presence of tapeta lucidum, which further enhance light sensitivity (83). Interestingly, although the tapetum was present in early vertebrates nearly 400 MYA, the ancestor of mammals probably lacked this structure. This is supported by the absence of tapetum in monotremes and the various types of tapeta found among other mammals and marsupials (88). Ungulates, whales and dophins share the fibrosal type, whereas dogs, cats, seals, bush babies and lemurs have a cellulosum tapetum (83, 88). That said, all tapeta have very similar mechanism of light reflection (T1 causation); increasing the amount of light absorbed by the photoreceptors in dim light.

## 8 Temporal vision—the ability to detect motions

As with color vision and spatial resolution, temporal resolution is typically sacrificed in dim light (85, 159). However, it pays to consider equine temporal resolution under daylight conditions. Interestingly, little research has been published in this area, even though motion perception is one of the most critical factors for ancestral and free-ranging horse survival. Indeed, as anyone with experience with horses will confirm, they are highly sensitive to moving object. While most dogs may be triggered to chase a moving object, most horses are more likely do treat it as a potential threat that should be attended to and avoided.

Temporal vision has often been appraised by presenting flickering light flashes which, at a certain frequency, become indistinguishable from steady light. Studies have shown that dogs can detect flickering light at 75 Hz, while humans reach 55–60 Hz [see review (90)]. Future studies will hopefully provide valuable insights into motion detection in horses, helping us to better understand both their ancestral predatory-prey interactions and their current responses to environmental stimuli today. Hence, without further research on horses, we will not delve deeper into Tinbergen’s questions. Instead, we will summarize common myths and misunderstandings about horse vision, before addressing the Five Domains (Table 1).

## 9 Implications for welfare—the Five Domains model

The Five Domains model for animal welfare assessment framework has been applied to sentient animals in research and teaching and is particularly valued for animals with which humans interact. It follows neatly from a consideration of Tinbergen’s Four Questions recognizing the dynamic relationships between affective state and biological function, and the importance of assessing both negative and positive animal welfare impacts of human behavior for each of the four physical domains: nutrition, physical environment, health, and behavioral interactions. We emphasize that the telos of horses determines what they value, how they detect potential harm, fear it and avoid potential harm. Like all animals, horses avoid, not just physical harm, but mental harms. The Five Domains framework ensures that anthropocentric priorities do not prevail over a species’ telos as one considers how each domain contributes to the animal’s mental state (i.e., the fifth domain) and the animals’ overall welfare state.

### 9.1 Domain 1: nutrition and hydration

During the first hours of a foal’s life, it is crucial that they find their dam and her udder. While olfactory and tactile cues are important in this challenge, vision may also contribute to a foal’s ability to locate milk. For example, it has been suggested that foals follow horizontal features to do so (91). Under natural conditions, this would likely be the ventral abdomen of the dam. Later in life, when differentiating its dam from other adult conspecifics, foals may use visual cues in combination with vocal recognition and olfaction. Furthermore, there is anecdotal evidence that domestic foals struggle to identify their dam when a human is on her back (McGreevy, pers. comm).

Visual information may help the horse to identify the most nutritious patches of grasses and forbs. Indeed, in other dichromatic herbivores, such as the swamp wallaby (*Wallabia bicolor*), both odor and visual cues can influence their browsing pattern (92). Horses have repeatedly been shown to prefer patches of long grass compared to intermediate and short grass (93, 94), suggesting that visual cues influence their foraging pattern when choice is available to them and conspecifics are not out-competing them. In contrast, studies on other ungulates, such as plains zebra (*Equus burchelli*), red hartebeest (*Alcelaphus buselaphus camaa*) and eland (*Tragelaphus oryx*) have found that foraging movement show little directionality, even when forage patches, such as palatable grass, are visible. However, they increased step length when moving toward non-visible patches. This suggests that these herbivores, living in large social groups, may not primarily rely on visual cues when foraging (95). That said, zebras showed an overall higher directionality in their movement compared to the red hartebeest and eland and might therefore be more efficient than these ruminants at locating new forage patches. These observational field studies must be viewed with some caution since we cannot know how, when surrounded by other appealing distractors and possible deterrents, the patches of grass appeared to the equids in question.

In a more controlled setting, a multiple preference test in cattle conducted in a 28-meter-long test arena revealed that individuals relied, to some extent, on visual cues when discriminating between forages, preferring green forage over dry light-brown, dead forage (165). Since the stimuli were rather small (25 × 25 cm), this indicates that cattle in everyday (non-experimental) situations may use vision to make this food choice at much larger distances. It is unclear what kind of visual cues they used because color, brightness and structure of the forage all differed but it is likely that these cues collectively influenced the animal’s choice.

Horses kept in enclosures appear to designate specific areas for defaecation and avoid grazing them, most probably as an evolved mechanism to avoid endoparasites, with the highest concentration of feces found at the center of these latrine area (96). In contrast, free-ranging horses do not graze and defaecate/urinate in separate areas (97). Therefore, there is probably no strong evolutionary pressure to avoid latrine areas using vision. However, this does not preclude the possibility that horses rely on visual and olfactory cues to avoid feces outside enclosures and to avoid latrines when confined to smaller enclosures.

### 9.2 Domain 2: physical and thermal environment

Most horses have emmetropic eyes (normal vision) although some breed-related differences have been reported (98–100). However, it should be noted that eye growth and refractive development can be affected when the eye does not receive clear visual input [see review (101)], which is particularly relevant for foals kept in stabled environments with limited visual stimulation. This outcome in foals merits consideration. In chicks, those with their eyes covered by translucent covers (blurring the retinal image) exhibited greater axial eye growth compared to control chicks and developed myopia (near-sightedness) within just a few days (167).

In addition, disruption of circadian rhythms, such as through constant exposure of light, may compromise eye development [see review (102)]. In humans, reduced melatonin production due to prolonged illumination has been linked to myopia in young people (103). In chickens, the circadian growth rhythm in the eye disappeared under continuous light but did not result in myopia (167), and Nickla (102) highlights that the relationship between light–dark exposure and eye development is complex, and that both constant light and darkness can disrupt normal ocular growth rhythms, even if they do not always lead to refractive errors. Therefore, for normal eye development, we should aim to provide foals with opportunities to view large visually rich environments. And, the use of constant lightning in housing systems should be carefully considered, especially for developing horses.

Vision may assist in avoiding wet and dirty areas to rest in the environment and avoid contact with obstacles, barriers and fences. As with avoiding latrine areas, other senses may be used to avoid unsuitable substrates on which to lie.

### 9.3 Domain 3: health and fitness

As mentioned earlier, good visual acuity and peri-orbital vibrissae may assist in sparing horses from eye injuries while browsing, for example, by enabling them to avoid penetrating objects such as thorns and barbs. Nevertheless, corneal ulcers are common among domestic horses, with one-third having a history of this condition (166). Interestingly, horses kept exclusively on pasture were reported to have a significantly lower prevalence than horses that were kept in a combination of pasture and stalls. Moreover, the seasonal or periodic use of fly masks was not linked to corneal ulcers.

In driven horses, blinkers help to maintain forward motion and avoid the risk of lateral distractions. For ridden horses, the use of blinkers and training methods such as hyperflexion that compromise vision, whether intentionally or otherwise, may reduce agency and autonomy of the horse and can thereby compromise welfare. It is proposed that military horses were forced into this posturer to render them less fearful of oncoming threats and therefore arguably safer for cavalry personnel (104). By the same token, blindfolding horses may reduce panic and allow them to be handled in an emergency (such as leading them out of a burning stable), but this recommendation has been challenged by evidence linking blindfolding to unpredictable behavior and elevated heart rates (105, 106). During handling tests in which horses (*n* = 33) were asked to move, navigate, or balance, blindfolded horses took longer to respond, required greater tension on the lead rope, and displayed more avoidance and refusal behaviors than non-blind-folded horses (105). Hence, in emergencies situations, blindfolding should be avoided.

What about protective goggles or plastic shields on racehorses? There are some historical pictures showing horses wearing some form of goggles, but the practice has gained more structure in modern times. In Japan, it is permitted to train racehorses with blinkers that include clear plastic cups over the eyes while galloping on wood-chip tracks. The practice is also beginning to appear in other parts of the world. Off the racetrack, there are also goggles including some UV-protectant varieties for horses with chronic uveitis whose eyes are sensitive to light. However, research on such devices is limited and further studies would be valuable, as such equipment could potentially help protect horses from eye injuries.

For the aging horse, it should be acknowledged that ocular diseases are common. Among a sample of domestic horses in Australian (*n* = 327) aged 15 years or over, 88% showed minor-to-severe disease (107). Similarly, and in the UK (*n* = 200), 94% of geriatric horses had at least one abnormality at ocular and ophthalmoscopic examination (108). As explained in this article, horses are highly visual creatures and their normal behavior depends on healthy vision. So, carers should be attentive to changes in horse behavior, especially shying when handled or ridden, and ensure veterinary ophthalmic examinations are deployed swiftly as part of consequent investigation.

Unilateral blindness could compromise the safety of personnel (not least in veterinary contexts) and affect responses to visual social and anthropogenic training cues. For example, a horse that is blind in one eye may miss visual training cues from the affected side and be inappropriately labeled wilful or disobedient, with a concomitant escalation of force. However, behavioral studies on horses with only one eye are scarce. A retrospective study by Utter et al. (109) showed that the majority (85%) of the horses (*n* = 77) that had one eye surgically removed returned to their previous discipline. However, our understanding of the visual consequences of unilateral enucleation in horses remains limited. In humans, spatial visual appears to be somewhat enhanced, while motion processing tends to be negatively affected [see review by González et al. (110)]. That review also highlight the brain’s plasticity following eye removal, where brain cells normally connected to the absent eye are recruited by the remaining one. Future studies should aim to clarify how animals with more laterally placed eyes than humans are behaviorally affected from unilateral enucleation.

### 9.4 Domain 4A: interactions with the environment

Domestic horses should be allowed to move freely to exhibit natural behaviors, preferably outdoors. Indeed, and in some countries, such as Sweden, this is a legal requirement (SJVFS 2021:30, Chapter 5 1§). However, the “outdoor environment” is rarely defined, either in terms of area or type of fencing. Some enclosures might have solid wooden fencing, while others use electrical tape fencing, or a combination of both. Still, we know relatively little about how horses perceive these barriers visually, even though white electrical tape fencing appears very bright compared to its surrounding, and appears to create strong visual contrast for human observers. However, at speed and particularly when aroused or frightend, horses may have difficulties noticing such barriers in time. Therefore, when introducing a horse to a new enclosure, it is advisable to walk them around the edges to help them familiarize themselves with the boundaries. It has been shown that horses (*n* = 20) avoid paddock boundaries more when surrounded by electrical fencing than by wooden fencing (111), although no differences were found in physiological or behavioral stress responses. It should be noted, however, that the two enclosures tested in this study were extremely small (2.25 m^2^ and 36 m^2^), so the presence of electrical fencing may not elicit the same equine responses in a larger space where the horses have more room to move.

Vigilance behavior, characterized by an elevated head position, is closely linked to antipredator responses and is frequently observed in Przewalski horses [*Equus ferus przewalskii*; (112)]. It has also been found to increase in domestic horses following exposure of predator vocalizations (113). Responses to novelty are commonly used to assess fearfulness, and in horses, an initial fearful reaction is typically found, both in behavior and physiological measures ((114), *n* = 24; (115), *n* = 18). Leiner and Fendt (115) also found behavioral responses adhered to a typical order, suggesting phases in the fear response. They generally start with the elongation of the upper lip followed by tension of the neck muscles which may be followed increases in heart rate, snorting and avoidance movements. Habituation to the novel stimulus reduced both the behavioral and physiological fear responses, but as the habituation process is stimulus-specific, exposure to a new novel object elicited a renewed fear response (115). Interestingly, Christensen et al. (114) found differing responses when testing novel objects of various modalities (notably vision, audition and olfaction). Both visual and auditory stimuli resulted in increased heart rate, whereas the olfactory stimulus did not. The behavioral responses to the visual and auditory novel objects were also similar, while the olfactory stimulus resulted in more sniffing behavior. However, a reduction in eating time was observed in response to all novel objects.

Individually stabled horses (*n* = 18) with no conspecific contact show an increase in their alert behavior compared to those with half walls that allowed close contact with neighboring horses (116). Additionally, both vigilance and stereotypic behaviors have been reported to be more frequent in frontally open stalls, where horses could put their head outside and view their surroundings, but had limited visual contact with only one neighbor, compared to enclosures with walls that provided both visual and some tactile social contact (117). Copper et al. (118) studied 10 horses, five of which had been known to weave (a stereotypic locomotory behavior) for at least 2 years, in different stable designs, and, similar to Lesimple et al. (117), they found more weaving when only the front half-door was opened. In contrast, horses in stables with a more open design, such as stalls with open backs or/and sides that allowed for greater social interaction, showed significantly less or no stereotypic behavior (118). Hence, if we accept that stereotypic horses are sentinels of challenges to telos for all horses (119), visually and socially restricted stable designs should be avoided to assure welfare for housed horses.

Horses that are able to observe their surroundings may be more settled than those with a limited view. This may explain why the amount of visual expanse stabled horses can see is inversely related to with the amount of sterotypic behavior (specifically weaving) they show (118). So, the traditional stable with four solid walls and a half-door, that may or may not be opened, is not aligned with optimal welfare.

As horses interact with novel elements within their environment, we may observe increased alertness as mentioned earlier. For example, when we ask the horse to enter a narrow trailer. In addition to being confined and carrying unfamiliar scents, the trailer walls also significantly reduce the horse’ panoramic view. Cross et al. (120) investigated how different lighting conditions in the trailer affected horses (*n* = 8) during loading. When the surroundings in which the horse was located were illuminated, the horses turned away more often whereas if the trailer was dark they sniffed the ground more. But regardless of illumination and contrasts, their heart rates and estimated stress levels increased during the loading process (120). However, a subsequent study by (168), reported fewer stress-related behaviors and shorter loading times when horses (*n* = 22) were loaded into trailers with interior LED lightning at high-intensity levels (above 4,500 lx) compared to those with lower levels. The authors speculated that the halogen lamps used in the earlier study by Cross et al. (120) may have been too dim for horses to see well in but also possibly created additional shadows, an effect that (168) deliberately avoided in their experimental set-up. The role of shadows in animal handling facilities always merits close attention, especially when animals stall for no apparent reason (121).

To avoid adverse experiences in horses, it is essential that they feel safe and have a sense of agency and some control over their choices. This can be achieved by the presence of familiar conspecific company that is comfortable with the environment and repeatedly associating challenging environments and situations with positive emotional experiences. For example, even at a young age, a foal may follow its mare into a trailer, finding security in her presence. As an adult, the presence of a conspecific can similarly help reduce stress in such challenging situations (122) especially when both can ingest food.

Interestingly, for isolated horses, even a mirror or a poster featuring a horse head may decrease stress-related behaviors, such as weaving in stables (123–125), and head-tossing and vocalization in the trailer (122). This highlights how even the mere perception of conspecifics can provide comfort, a topic we will come back to in the 4B Domain. In a similar vein, the sight of hay (or other valued food) offered repeatedly in association with entering the trailer can create a positive affective state when loading. For example, Dai et al. (126) showed significantly reduced loading times using a target training program with positive reinforcement, where three horses were trained to self-load. Additionally, in a related study, although the sample size was again small (*n* = 6), Yngvesson et al. (127) found that loading time was halved on the second training day, during which both positive and negative reinforcement protocols were used. While the current review does not delve into learning theory or specific training strategies, it is important to emphasize that there are biologically salient ways to deploy visual cues to help horses feel at ease, even in challenging situations. Understanding how best to present such cues requires an appreciation of the visual biology of horses, as summarized earlier in this article. There are avenues for future research that explore optimal representations of conspecifics and even the deployment of so-called super stimuli [i.e., where visual attributes of a stimulus are enhanced to evoke persistent responses that are desirable; (128)].

The ground that we ask horses to tread on can have visual properties that horses find aversive. Clearly, many horses react to puddles in ways that suggest that they do not perceive them as merely shallow pools of water. This is intriguing, but prompts one to recall that puddles often reflect the sky or surroundings, making them appear shiny, moving, or even potentially fathomless to a horse. This visual effect can startle them. Equally, the glare generated by the horizontally polarized light, as it is reflected by the water’s surface, may also transiently dazzle them. And even though we know the horse has the ability for depth perception, a reflective surface can obscure the true depth of water, making it appear potentially dangerous. The same goes for muddy water, where sediment defies an appreciation of the ground below. In a similar vein, it has been shown that horses react more to unexpected colors and glossy whites and blacks on the ground than to more natural colors, such as green and gray ((129); *n* = 16) even more so than when these stimuli are presented on the wall. However, a significant habituation effect was observed over repeated tests highlighting the importance of familiarity and exposure in reducing visual reactivity. Furthermore, Saslow (130) found that both stimulus size and contrast against the ground influenced the behavioral response of approaching horses (*n* = 11). Notably, one-third of the horses did not react at all to the smallest stimulus (1.27 cm) when it had low contrast, even as they passed over it. This serves as a reminder that humans experience their surroundings differently from horses and highlights the benefits of personnel applying knowledge of equine visual biology to help horses and allow them time to move their heads and change their posture as they develop confidence in approaching visual challenges in similar challenging situations.

### 9.5 Domain 4B: interaction with other animals

For horses, their social group represents safety, and being within it is closely linked to survival. This underlines the importance of social contact and interactions, where their high visual acuity plays a crucial role. Indeed, horses exhibit a rich and nuanced body language with subtle variations, such that even cues from the ears and eyes are sufficient for effective communication with conspecifics (53, 54). Recently, Lewis et al. (131) carefully analyzed their rich repertoire of facial movements and previous research (54) has shown that distinct facial expressions in horses can elicit different responses from conspecifics. Given horses’ relatively high visual resolution, future studies could explore how horses respond to varying degrees of face expressions in conspecifics, particularly whether they are able to detect and react differently to subtle versus more pronounced “pain faces” in bonded and unfamiliar conspecifics.

The importance of social bonds is repeatedly confirmed by physiological evidence. For example, Hartmann et al. (132) found that during training involving social separation, horses showed significantly lower heart rates when a familiar companion was present (*n* = 32). However, even those initially trained in pairs exhibited increased heart rates during later separations. Furthermore, a companion horse can significantly reduce a horse’s behavioral response to a visually novel object, such as a large striped ball (133). Although the immediate reaction to a rapidly opened umbrella was similar whether the horse was tested alone or in pair, the heart rate recovery was significantly faster when the horse was together with a companion horse (*n* = 32, 16 pairs).

Social contact with conspecifics is essential for equine welfare. It is known that horses rarely lie down as a group. Being able to see other horses grazing or standing may offer cues to horses that allow them to feel comfortable adopting recumbent positions. Furthermore, as mentioned earlier, when isolated in the stable, horses have been shown to respond positively to mirrors, suggesting that such biologically salient visual reflections may, in the short-term at least, help alleviate feelings of separation from actual conspecifics (122–124). Similarly, in other domesticated ungulates, cattle have repeatedly demonstrated reduced stress when isolated with mirrors or images of conspecifics (169). The comfort of conspecific company is associated with a positive affect and feeds into good overall welfare (D5). So, for horses that are physically isolated, being able to see others may act as form of partial compensation.

### 9.6 Domain 4C: interaction with humans

Interestingly, in some contexts, even humans can provide social buffering for horses. In a separation-reuinon experiment, Lundberg et al. (134) found that horses sought human proximity during the reunion phase and had significantly lower heart rates in the presence of both their owner and a stranger, compared to a complete separation phase. This highlights the stress horses experience when left vulnerable and alone. Even though they behaved similarly toward both the owner and the stranger during a reunion (134), it is likely that, if given the choice, they would prefer the presence of a conspecific.

Vision is one of the primary shared information channels in horse-human interaction, allowing us to gather information from each other’s body language, even from a considerable distance. In human-horse interactions, it is well established that human posture and attentiveness influence the behavior of the horse. Research has shown that horses prefer attentive humans, such as those facing them rather than looking away, or those with their eyes open rather than closed (46). Indeed, some horses have been shown to take detours to seek eye contact if the human was facing away. Regarding human posture, horses struggle to recognize humans adopting a quadrupedal stance (McGreevy, pers. comm) and may prefer those adopting a submissive (longitudinally flexed) body posture over dominant (extended) body postures (135). This reflects how attentive and skilled horses are when reading human body language. How they respond to this information seems to depend on their previous experiences with humans, as research has shown that horses trained with positive reinforcement, even for just a couple of weeks, seek more contact with humans than those trained using traditional negative reinforcement alone (134, 136).

Not surprisingly, the ways in which we approach horses affects their behavioral responses. A fast approach increases the flight distance compared to a slower one, and swinging a rope can triple the flight distance compared to a less aversive approach [(137), *n* = 54]. However, this reaction may not be purely visual as it could also be influenced by the sound of the swinging rope. Regardless, this should be considered in the same way as using a lunging whip, as one wants to avoid unnecessarily arousing or frightening the horse, since a fearful horse is more likely to react unpredictably, compromising both welfare and safety. Along similar lines, we have already noted that horses respond to novelty in various modalities. Therefore, we should be mindful of how our actions appears to nearby horses when performing sudden actions such as removing a jacket, especially while mounted, as most novel visual and auditory stimuli could be perceived as a threat.

During handling, it has been suggested that horses may be more compliant when the human is on one side rather than the other. This could reflect traditional left-biased training methods or sensory lateralisation. Sensory lateralisation is thought to be linked to lateralized processing in the brain of various emotional stimuli, with the two hemispheres specializing in different information. For example, horses have been shown to react more strongly to suddenly opened umbrellas when these appear in the left monocular field, indicating right hemisphere activation, which is often linked to flight responses (138). The stronger estimated emotional reaction, the more a horse will look at a novel object with its left eye (18, 19). However, it is worth noting that in Baragli et al. (20), a similar number of horses showed a preference for the left and right eye when observing an inflated novel balloon. The authors speculated that individual subjective emotions may have varied, with some horses being more curious than fearful.

Farmer et al. (139) tested visual laterality in horses (*n* = 55) in four experiments, both in the presence of a passive familiar human and a stranger and during training interactions with a familiar handler. This study showed that, in general, most horses revealed a left-eye preference when looking at the passive person. This bias was stronger in traditionally trained horses compared to those trained from both sides (139) but interestingly the difference between the groups of horses disappeared during the interaction test. Thus, horses appear to have a left-eye preference in their interactions with humans, regardless of how familiar they may be, and Farmer et al. (139) suggest that this preference for keeping people to their left is linked to emotion processing, rather than habit and training. The use of the left eye, which is typically associated with the processing of strong emotional stimuli regardless of their valence (see review 170), was therefore suggested to reflect heightened emotional arousal, potentially underlying motivations such as a desire to cooperate or, alternatively respond quickly (139). Indeed, Karenina et al. (140) found a left-side bias in mother-infant interactions across several mammalian species, including horses, suggesting that the right hemisphere is involved for positive as well as negative emotions. It is interesting to speculate that this population bias toward left-sided social interactions may explain why, beyond merely inculcating consistency which in itself assists in training (141), traditional horse-handling practices, military drills and equipment design favor approaching from the horse’s left (142).

During equitation, horses (*n* = 12) showed more behavioral indicators of stress when ridden in an arena and exposed to a moving and talking audience (or moving-only audience) compared to when no audience is present (143). These indicators were accompanied by both increased salivary cortisol concentrations, indicating activation of the hypothalamic–pituitary–adrenal axis, and lower heart rate variability, reflecting autonomic nervous system activity. Horses can, of course, become accustomed to an audience, but it should be acknowledged that additional visual input an audience presents during riding may heighten the horse’s arousal and perception of novel stimuli, potentially compromising their sense of safety and reducing their focus on cues from the rider. Therefore, during any attempts at habituation to crowds, the demands personnel place on horses should be adjusted to allow them to adapt to the increased visual challenges as the numbers in the audience increase. Associating novel visual stimuli, such as obstacles that may appear in show-jumping, with resources such as calm conspecific company and food may help horses to find them less threatening.

Clearly, a central part of the challenge in competitive jumping is training horses to overcome their innate preference to avoid novel stimuli, largely because they have evolved to value a clear view of the ground ahead of them. In equestrian sports, particularly show-jumping, the colors of obstacles are often discussed as previously outlined by Rørvang et al. (2). Indeed, research has shown that fence color affects how the horses jump. For example, horses take off from a greater distance when approaching a white fence compared to an orange one, as white provides a sharper contrast against green or brown backgrounds, whereas orange blends in more due to horses’ dichromatic vision (144). Observations from show-jumping competitions further reveal that single-colored obstacles result in more faults than those with two contrasting colors (145), confirming the importance of both color and intensity contrasts.

Rørvang et al. (2) also highlighted the persistent trend in dressage where horses are ridden with over-flexed necks, “behind the bit,” which, while potentially linked to compromised pulmonary ventilation and vertebral issues, significantly limits the horse’s vision and may thus increase stress levels. In this vein, a recent comprehensive study by Kienapfel et al. (146) reports significantly more conflict behaviors in elite dressage horses when ridden with their nasal plane behind the vertical. Alarmingly, most of the horses in their study (178 out of 191) were ridden “behind the bit,” confirming serious welfare concerns in the sport (147, 148), especially at the higher levels where this flawed frame seems to attract better scores (149). Addressing these issues is essential to improve the legitimacy of the discipline and to align practice with scientific research.

### 9.7 Domain 5: the mental state of the horse

It is clear that vision is critical for survival in equids. So, it is appropriate to describe horses as visual beings. The telos of a horse explains why being able to survey both their surroundings for potential threats and neighboring conspecifics for subtle signals matters to them. Any event or outcome that challenges horses’ telos by compromising their vision and ability to assess the potential risks can cause them distress ranging from fear to pain. For this reason, in the absence of habituation and associative learning, unhandled horses generally exhibit avoidance of humans as they do all novel visual stimuli, treating them as potential threats.

As discussed above, all of the physical domains that can present visual challenges to horses are expected to have negative effects on the mental state of horses. Additionally, impairments such as eye injuries, restricted visual fields due to equipment or environmental limitation (such as solid-walled stables that obstruct sight, or poorly lit trailers) can increase uncertainty and vigilance in horses. This heightened alertness may create distress, more frequent sudden fear responses, and less safe horse-human interactions. Therefore, safeguarding the horses’ visual awareness, prioritizing their sense of agency and avoiding sudden visual challenges should enhance their overall mental state.

However, we should not be content with merely avoiding compromised welfare, or ensuring only good physical health. Instead, we should aim to promote positive welfare for horses. A recent definition by Rault et al. (150) highlights that “*positive animal welfare is defined as the animal flourishing through the experience of predominantly positive mental states and the development of competence and resilience*.” In the future, this may be assessed using non-invasive evidence such as detailed ethograms of their facial expressions linked to differing emotional valence (131). Emerging findings also suggest that emotional states, and thereby welfare, in horses can be linked to specific behaviors. For example, the half-blink has been proposed as an affiliative facial gesture (151). These developments provide promising insights for advancing welfare assessments.

In the crudest sense, the mental and emotional state of the horse (D5) represents the summed subjective experience arising from all other domains. In parallel, the interpretation of ambiguous cues in a judgment bias test (152, 153) holds enormous promise in evaluating equine welfare. Typically, in judgment bias testing, the animal is trained with one positive stimulus and one negative stimulus, and then tested using one or more unrewarded ambigious cues to assess whether it perceives them as more like the positive (“glass half full”) or negative (“glass half empty”) stimulus—indicating a more optimistic or pessimistic bias, respectively. However, as Mendl et al. (153) summarize, the methods of cognitive/judgment bias testing can vary, and each approach has its own advantages and disadvantages that may influence the outcome and its relevance to the animals’ immediate and long-term welfare state. With such variability in play, the influence of vision and lighting on how these tests are presented to horses is therefore currently inestimable.

In horses, Henry et al. (154) found that living and working conditions had a strong effect on performance in a judgment bias test. They compared horses from two riding schools (RS; *n* = 25) that were housed in single boxes, ridden by inexperienced riders, and received limited roughage with horses from two leisure sites (LS; *n* = 9), where horses lived in stable social groups, had access to free-range, and were used for leisure riding. The results were in line with their hypothesis that the RS horses displayed a more pessimistic response toward an ambiguously located stimulus than the LS horses. That said, the authors emphasized that living conditions can vary considerably within both RS and LS contexts and may even be reversed. For instance, some RS horses may experience more favorable conditions than some LS horses, depending on how either context is managed. Nevertheless, their findings highlight the importance of ensuring positive living conditions and equestrian practices since they found clear association between pessimistic bias and indicators of reduced welfare. Hence, cognitive judgment bias may be a valuable tool for assign emotional state in horses, just as detailed ethograms of their facial expressions can be.

## 10 Conclusion

To conclude, research has debunked many common beliefs about horse vision. Horses possess visual adaptations that enables them to perceive fine details, see colors and maintain good vision in low-light conditions. Indeed, vision is a critical sense for horses. However, their evolutionary history as a prey species has shaped them to be cautious toward novel shapes and movements, as these may signal potential threats. Gaining a better understanding of how horses perceive the environments we impose on them, particularly under varying light conditions, is not just beneficial, but essential for improving their welfare and promoting safer human-horse interactions.
